# Tree shrews as a new animal model for systemic sclerosis research

**DOI:** 10.3389/fimmu.2024.1315198

**Published:** 2024-01-26

**Authors:** Leting Zheng, Shuyuan Chen, Qiulin Wu, Xi Li, Wen Zeng, Fei Dong, Weiwei An, Fang Qin, Ling Lei, Cheng Zhao

**Affiliations:** ^1^ Department of Rheumatology and Clinical Immunology, the First Affiliated Hospital of Guangxi Medical University, Nanning, China; ^2^ Department of General Surgery, the Second Affiliated Hospital of Guangxi Medical University, Nanning, China; ^3^ Key Laboratory of Clinical Laboratory Medicine of Guangxi Department of Education, Department of Clinical Laboratory, the First Affiliated Hospital of Guangxi Medical University, Nanning, Guangxi, China; ^4^ Respiratory and Critical Care Medicine Department, the First Affiliated Hospital of Guangxi Medical University, Nanning, Guangxi, China

**Keywords:** tree shrew, systemic sclerosis, animal model, inflammation, fibrosis, RNA sequencing

## Abstract

**Objective:**

Systemic sclerosis (SSc) is a chronic systemic disease characterized by immune dysregulation and fibrosis for which there is no effective treatment. Animal models are crucial for advancing SSc research. Tree shrews are genetically, anatomically, and immunologically closer to humans than rodents. Thus, the tree shrew model provides a unique opportunity for translational research in SSc.

**Methods:**

In this study, a SSc tree shrew model was constructed by subcutaneous injection of different doses of bleomycin (BLM) for 21 days. We assessed the degree of inflammation and fibrosis in the skin and internal organs, and antibodies in serum. Furthermore, RNA sequencing and a series of bioinformatics analyses were performed to analyze the transcriptome changes, hub genes and immune infiltration in the skin tissues of BLM induced SSc tree shrew models. Multiple sequence alignment was utilized to analyze the conservation of selected target genes across multiple species.

**Results:**

Subcutaneous injection of BLM successfully induced a SSc model in tree shrew. This model exhibited inflammation and fibrosis in skin and lung, and some developed esophageal fibrosis and secrum autoantibodies including antinuclear antibodies and anti-scleroderma-70 antibody. Using RNA sequencing, we compiled skin transcriptome profiles in SSc tree shrew models. 90 differentially expressed genes (DEGs) were identified, which were mainly enriched in the PPAR signaling pathway, tyrosine metabolic pathway, p53 signaling pathway, ECM receptor interaction and glutathione metabolism, all of which are closely associated with SSc. Immune infiltration analysis identified 20 different types of immune cells infiltrating the skin of the BLM-induced SSc tree shrew models and correlations between those immune cells. By constructing a protein-protein interaction (PPI) network, we identified 10 hub genes that were significantly highly expressed in the skin of the SSc models compared to controls. Furthermore, these genes were confirmed to be highly conserved in tree shrews, humans and mice.

**Conclusion:**

This study for the first time comfirmed that tree shrew model of SSc can be used as a novel and promising experimental animal model to study the pathogenesis and translational research in SSc.

## 1 Introduction

Systemic sclerosis (SSc) is a chronic systemic disease characterized by immune dysregulation and the development of cutaneous and visceral fibrosis (1). The prevalence of SSc is estimated to be approximately 1 in 10,000 individuals (2). Despite its relatively low incidence, SSc stands as one of the rheumatic diseases associated with the highest mortality rates, primarily due to the involvement of vital organs such as the lung, heart, digestive tract, and kidney (3). Notably, the 5-year survival rate for SSc patients is reported to be 74.9%, which significantly declines to 40% in cases where visceral organ damage occurs (4, 5). Moreover, the incidence of malignancy was significantly increased in SSc patients (6). Therefore, SSc imposes a substantial financial burden on patients and their families (7, 8). Despite extensive research efforts, the etiology and pathogenesis of SSc remain elusive, and effective therapeutic interventions for this disease are currently lacking. Consequently, it is of utmost importance to investigate the potential mechanisms underlying SSc progression, as such insights hold significant implications for the diagnosis and treatment of this debilitating condition.

An optimal animal model holds significant value in investigating the etiology and pathogenesis of SSc and in the development of novel therapeutic interventions. Currently, mouse models serve as the primary animal models for SSc resarch, employing various approaches such as exogenous drug administration and genetic engineering (9–11). Among the induced models, the bleomycin (BLM)-induced SSc mouse model stands as the classical model due to its ease of implementation, cost-effectiveness, and high success rate in modeling. This model primarily focuses on the inflammatory response and tissue fibrosis characteristic of SSc, while comparatively neglecting the immune response and vascular pathology associated with the disease. Genetic models, on the other hand, exhibit advantages in simulating tissue fibrosis and vasculopathy in SSc, compensating for the limitations of induction models to some extent. However, the technical intricacy and elevated costs associated with genetic models restrict their widespread application. Although mouse models play an essential role in elucidating the mechanisms underlying SSc and facilitating drug development, it is important to acknowledge that mice and humans have divergent origins, resulting in notable disparities in physiological and immunopathological characteristics. Consequently, the translation of findings from animal models to clinical applications is somewhat limited. This limitation is exemplified by the failure of certain antifibrotic drugs, which demonstrated efficacy in mouse SSc models, to yield positive outcomes in clinical trials (12, 13). Nonhuman primates, given their closer anatomical, physiological, and genetic resemblance to humans, are frequently employed in the study of human diseases. However, ethical concerns, high costs, and lengthy reproductive cycles have hindered their widespread utilization. Therefore, it is imperative to explore alternative animal models that closely resemble humans, possess ease of reproduction, and faithfully replicate the clinical manifestations of human SSc.

The tree shrew (Tupaia belangeri), a diminutive mammal resembling squirrels in physical appearance, exhibits a geographical distribution primarily encompassing South Asia, Southeast Asia, and the Yunnan and Guangxi regions of China (14). Through whole-genome sequencing, it has been ascertained that tree shrews exhibit a closer transcriptome similarity to primates than mice across four mammalian tissues, including those from humans, macaques, tree shrews, and mice (14, 15). In contrast to mice and other rodents, tree shrews demonstrate a remarkable resemblance to primates in terms of taxonomy, anatomy, physiology, biochemistry, neurodevelopment, immunology, and other biological characteristics. Consequently, tree shrews have emerged as promising small mammal models that hold potential for replacing primates in disease research (16, 17). Furthermore, tree shrews possess several advantageous traits, including their small size, short reproductive cycle and lifespan, and low feeding costs, thereby combining the benefits of both primates and rodents (18). Notably, tree shrews have been extensively employed in the establishment of disease models for viral hepatitis, neuropsychiatric disorders, metabolic ailments, visual impairments, and cancers, thereby showcasing unique advantages that have garnered recognition across various medical disciplines (19–23). In recent years, researchers have increasingly turned to tree shrews as a valuable resource for constructing animal models of autoimmune diseases, successfully establishing models for rheumatoid arthritis and systemic lupus erythematosus in tree shrews (24, 25). However, to date, no studies have explored the development of a tree shrew model for SSc. Notably, the anatomical structure of tree shrew skin bears striking resemblance to that of human skin (26), suggesting that the tree shrew holds promise as a novel animal model for investigating SSc.

To gain a deeper understanding of genes and their involvement in various biological processes, gene sequencing and bioinformatics methods have become indispensable tools in molecular biology and genetics research (27–29). Recently, the application of RNA sequencing has facilitated the investigation of the transcriptional profile of brain tissue in the tree shrew model of diabetes complicated with ischemic stroke (22), as well as the transcriptional landscape of the liver in the tree shrew model of diabetic liver disease (30). However, no relevant study has explored the transcriptome sequencing of tree shrew skin.

In this study, we successfully established a tree shrew model of SSc for the first time. This SSc tree shrew model exhibited inflammation and fibrosis in skin and lung, part of the animals developed esophageal fibrosis and autoantibodies, including antinuclear antibodies (ANA) and anti-scleroderma-70 (anti-Scl-70) antibody, all of which close to the pathological characteristic of human SSc. Using RNA sequencing, we anlysed the transcriptome profiles and immune cell infiltration in the skin of SSc tree shrew models, and controls, and identified hub genes through a series of bioinformatics analyses. Our study offers a novel and promising experimental animal model to study the pathogenesis and translational research in SSc.

## 2 Materials and methods

### 2.1 Animal grouping and SSc modeling

Forty-four adult tree shrews of both sexes (10-12 months old, weight 120-140 g, production approval number: SCXK (Dian) 2020-0004) were provided by the Experimental Animal Center of the Kunming Institute of Zoology, Chinese Academy of Sciences. Animals were housed in individual cages at a room temperature of 23 ± 2°C and a 12/12-hour light/dark cycle at the Primate Experimental Center of Guangxi Medical University with free access to water and food. The protocol of the present study was approved by the Experimental Animal Ethics Committee of Guangxi Medical University (No. 20180521).

The concentration of BLM administered was determined using the body surface area method of dose conversion (31, 32). Prior to the experiments, adaptive feeding was conducted for a duration of two weeks. Thirty-five tree shrews were randomly allocated into four groups, including the control group (n=8), low-dose (n=9), medium-dose (n=9), and high-dose (n=9) BLM-induced SSc model group. The optimal BLM concentration was determined after model evaluation. Sequently another 9 animals were randomly divided into control (n=4) and SSc model group which was induced by optimal BLM concentration (n=5) for skin RNA sequencing. In the model groups, the animals received daily subcutaneous injections on their shaved upper back (2 cm × 2 cm) for a period of 21 days. The injection consisted of 100 µl of BLM solution with concentrations of 0.4 mg/ml (low-dose group), 2 mg/ml (medium-dose group), or 4 mg/ml (high-dose group). Shaving of the upper back was repeated weekly or as required. In contrast, the control group received daily subcutaneous injections of 100 µl of phosphate buffered saline (PBS) for 21 days. Throughout the experiment, the health status and changes in skin lesions of the animals were monitored on a daily basis, and their weight was recorded weekly. On the day of the final BLM injection, the animals were sacrificed, and their serum, skin, lungs, esophagus, and kidneys were collected for subsequent analyses.

### 2.2 Histopathology

The harvested skin, lungs, esophagus and kidneys were fixed and paraffin-embedded. Paraffin tissue sections (4 μm) were stained with hematoxylin and eosin (HE) and Masson’s trichrome. The Olympus pathological image analysis system software was utilized to measure the skin thickness. The collagen volume fraction (CVF) of the skin tissue was quantified and analyzed using ImageJ software. The skin inflammation scores were determined by employing a semiquantitative scoring system, as previously described, based on the examination of five microscopic fields. The scoring system ranged from 0 to 4 (0, none; 1, little; 2, mild; 3, moderate; and 4, severe) (33). The lung inflammation scores were assessed by counting leukocytes in the perivascular, interstitial, and peribronchial areas, with scores of 0 (scattered), 1 (5-15 leukocytes/field), 2 (15-50 leukocytes/field), and 3 (>50 leukocytes/field) (34). The average inflammation score from five different fields was calculated. The Ashcroft lung scores, which range from 0 (normal lung) to 8 (complete fibrosis), were determined by averaging scores from five microscopic fields (35). All measurements and scores were performed by two observers who were blinded to the group assignments.

### 2.3 Hydroxyproline assay

Skin and lung fibrosis are characterized by the excessive accumulation of collagen. Hydroxyproline is a major component of collagen. The quantification of hydroxyproline content in skin and lung tissues serves as a reliable indicator of collagen deposition and enables the evaluation of fibrosis severity. The measurement of hydroxyproline content was conducted following established protocols. In brief, 50 mg of skin and lung tissue was homogenized in saline and subsequently hydrolyzed in 2 N NaOH at 95°C for 20 minutes. The pH of the solution was adjusted to 6-6.8, and distilled water was added to achieve a final volume of 10 ml. After centrifugation, the resulting supernatant was prepared for hydroxyproline content measurement. The optical density (OD) value at a wavelength of 550 nm was converted to the corresponding hydroxyproline content (mg/g).

### 2.4 Measurement of α-smooth muscle actin in the skin

Immunohistochemistry was employed to detect α-Smooth muscle actin (SMA) in skin tissue. To elaborate, paraffin-embedded tissue sections were subjected to deparaffinization through preheating, xylene deparaffinization, and rehydration. Subsequently, antigen retrieval and blocking were carried out. The tissue sections were then incubated overnight at 4°C with primary antibodies (rabbit anti-α-SMA antibody, Cell Signaling Technology, diluted at 1:500). Following this, secondary antibodies were applied for 15 minutes at room temperature in the absence of light. The tissue sections were further treated with DAB for 5 minutes and counterstained with hematoxylin. Quantitative analysis was performed using the Olympus image acquisition system and ImageJ software analysis system. Ten nonoverlapping fields (400×) were randomly selected to measure the cumulative optical density (IOD) and positive area (area) in each field. The average optical density (AOD) value was calculated as AOD=IOD/area. The AOD value obtained from the measurement of 10 sections was considered as the representative AOD value for the respective sample. Two independent observers conducted image reading and measurements.

### 2.5 Detection of serum antinuclear antibody and anti-Scl-70 antibody

Serum anti-nuclear antibodies (ANA) was detected by indirect immunofluorescence assay on HEp-2 cell substrates following the manufacturer’s instruction and the initial dilution was 1:100 (EUROIMMUN Medizinische Labordiagnostika AG). The ANA partern was judged according to the International Consensus on ANA Patterns (ICAP). Photographs were taken by the LED fluorescence microscope EUROStar III Plus at 495 nm (green). The measurement of serum anti-Scl-70 antibody was conducted by enzyme-linked immunosorbent assay (ELISA). The mouse anti-Scl-70 ELISA kit was procured from Shanghai Yubo Biotechnology Company. All experimental procedures were meticulously performed in strict accordance with the instructions provided with the respective kits.

### 2.6 RNA sequencing

A total of 9 skin samples were collected for RNA sequencing: 5 skin samples with fibrotic lesions from SSc tree shrews models induced with medium-dose BLM and 4 from controls. In brief, the isolated skin tissues were promptly frozen in liquid nitrogen to prevent RNA degradation. Total RNA extraction was performed using the TRIzol reagent kit (Invitrogen, Carlsbad, CA, USA). Subsequently, the mRNA was subjected to enrichment, while the rRNA was effectively eliminated. The enriched mRNA was subsequently subjected to reverse transcription, resulting in the generation of complementary DNA (cDNA). The cDNA was purified, followed by the addition of poly(A) tails, and subsequent ligation to Illumina sequencing adapters. The resulting ligation products were subjected to size selection, PCR amplification, and ultimately sequenced using the Illumina NovaSeq 6000 platform.

### 2.7 Data processing and DEG screening

For background correction, normalization among the arrays of 9 skin samples, and differential expression analysis, the limma package in R was utilized. The cutoff thresholds for differentially expressed genes (DEGs) were samples with |log fold change (FC)| > 2 and an adjusted false discovery rate P <0.05. The DEGs are displayed in the heatmap and volcano plot.

### 2.8 Functional enrichment analysis

The Gene Ontology (GO) terms, which consisted of molecular function, cellular component, and biological process, were analyzed. ClusterProfiler (http://www.bioconductor.org/packages/release/bioc/html/clusterProfiler) was used to determine whether GO keywords were highly enriched with respect to the DEGs during GO analysis. GO words identify the biological processes, molecular activities, and cellular components to which a certain set of genes is most closely related. The clusterProfiler R program was used to conduct the pathway enrichment analysis for KEGG, with a significance level of p<0.05. ClusterProfiler was used to determine whether KEGG pathways were highly enriched in relation to the differentially expressed genes during the KEGG study. The data provided by KEGG pathways can be used to elucidate the molecular networks and interactions that underpin a wide range of biological activities.

### 2.9 Immune infiltration analysis

The CIBERSORT algorithm (36) was utilized to calculate an estimate of the proportion of immune cells that are present in involved skin tissues of control and BLM-induced SSc models. The method builds a machine learning-based model using known gene expression profiles of immune cell subsets. It then compares the gene expression data of a sample to the model to infer the relative proportions of different immune cell subsets within that sample. R’s “corrplot” tool was utilized for correlation analysis as well as visualization of the 22 distinct types of immune cells that had penetrated the tissue. The “vioplot” package in R was used to generate violin plots to illustrate the differences in infiltrated immune cells that were found between the two groups. Correlations between immune cell infiltration were evaluated via Spearman correlation analysis.

### 2.10 PPI network construction

The PPI and molecular function networks of the DEGs were built using STRING. We used Cytoscape (https://cytoscape.org/) to depict the PPI network and the plugin Molecular Complex Detection (MCODE) to determine whether the heavily linked modules were molecular complexes or clusters.

### 2.11 Conservation analysis of genes

To analyze the conservation of tree shrew genes, the target gene sequence data of tree shrew, human and mouse were collected from public databases. Multiple sequence alignment analysis was used to analyze the conservation of the selected target genes in tree shrews, humans and mice.

### 2.12 GeneMANIA

Protein interaction prediction and analysis of coexpression, colocalization, pathways, and physical interactions were performed using GeneMANIA (http://www.genemania.org). The possible protein functions of genes were investigated using website prediction and other profiles.

### 2.13 Statistical analyses

All statistical analyses were performed using SPSS statistical software version 26.0 (SPSS Inc., Chicago, IL, USA). All data were analyzed using one-way ANOVA for differences between groups, comparisons between groups with homogeneous variance were made using the Least-significant difference (LSD) method, and those with heterogeneous variance were analyzed using the Games-Howell method. Data are shown as the mean ± SD. A *P* < 0.05 was considered statistically significant.

## 3 Results

### 3.1 Safety of tree shrew SSc model induced by subcutaneous injection of BLM

**Figure 1:**
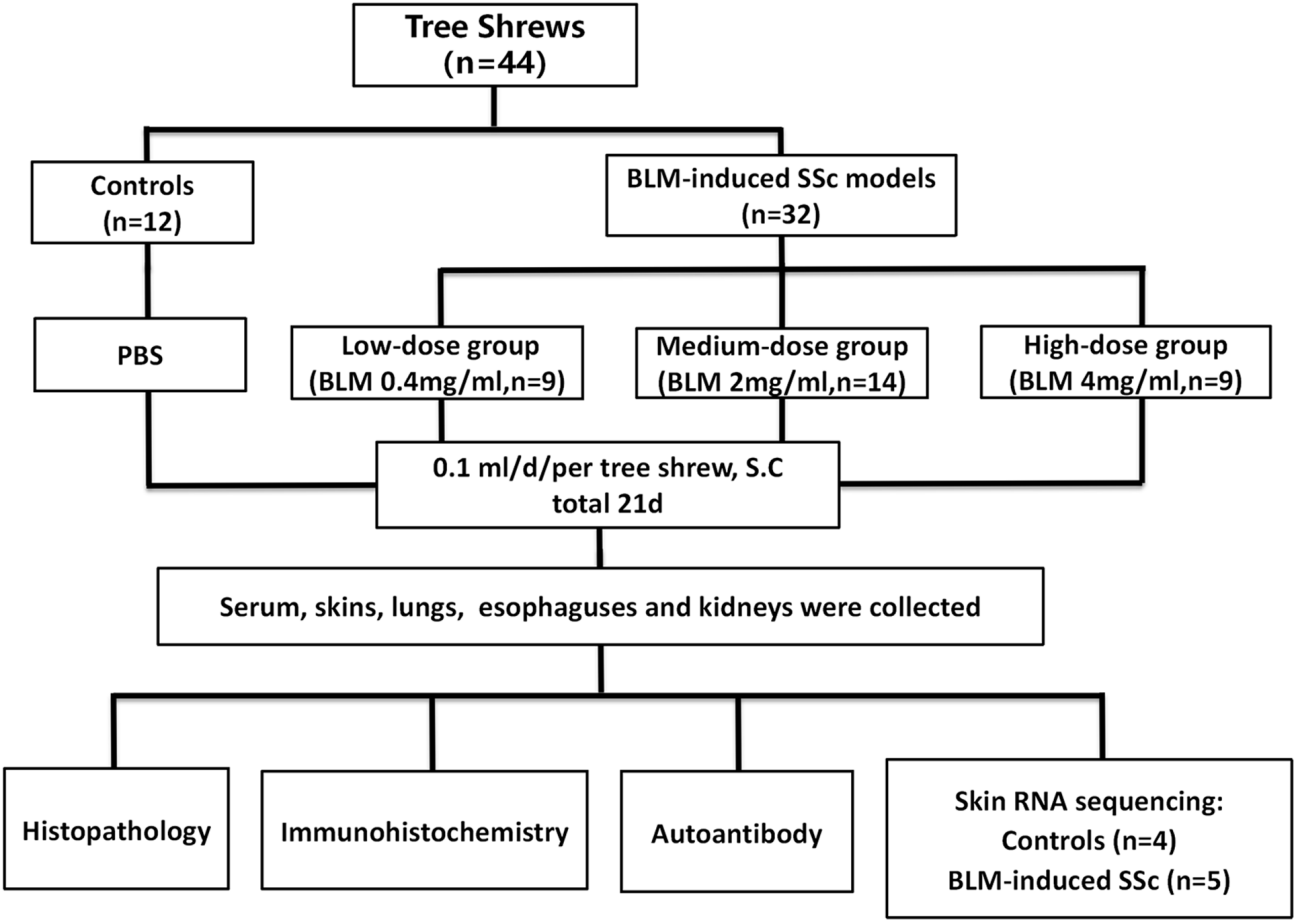
Workflow of this study.

The overall process of tree shrew SSc modeling is shown in **Figure 1**. When treated with BLM, some tree shrews exhibited reduced food intake and activity, but no significant changes in body weight compared with the controls. Throughout the modeling period, 2 out of 8 tree shrews in control group escaped, 2 out of 9 tree shrews in low-dose BLM group died, 2 out of total 14 tree shrews in medium-dose BLM group died. No animals in the high-dose BLM group died, but ulcers in the skin injection site were common. Overall, the subcutaneous injection of BLM was safe for tree shrew SSc modeling.

### 3.2 Histologic assessment of the BLM-induced SSc tree shrew model

Pathologic evaluation of the SSc tree shrew model was conducted 21 days of BLM treatment. Overall, our findings showed that subcutaneous injection of BLM in tree shrews successfully induced inflammation and/or fibrosis in the skin and internal organs.

#### 3.2.1 Skin

**Figure 2:**
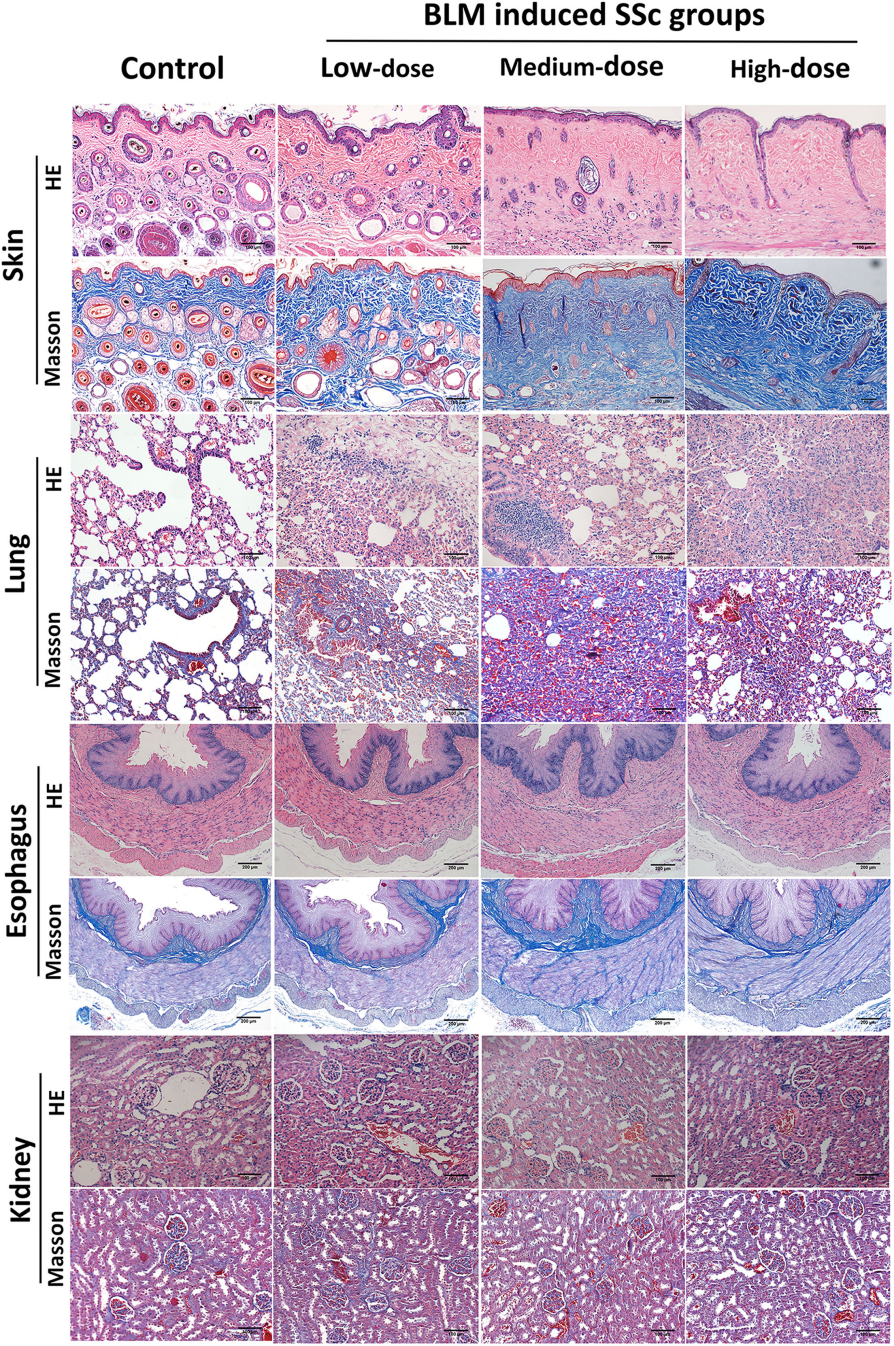
Pathological changes in various organs of tree shrews. Subcutaneous injection of BLM successfully induced a tree shrew SSc model to mimic human SSc inflammation and fibrosis characteristics. Representative skin, lung, esophagus and kidney histologic sections in each group of tree shrews stained with hematoxylin and eosin (HE) and Masson’s trichrome (original magnification ×200 for skin, lung and kidney; original magnification ×100 for esophagus) are shown.

No histologic changes were found in the skin of control group. The low-dose BLM group showed mild thickening of the dermis, slight infiltration of inflammatory cells and mild increase in collagen fibers. In the medium- and high-dose BLM groups, tree shrews exhibited marked thickened dermis, infiltration of inflammatory cells and increased collagen fibers. Skin inflammation score and hydroxyproline content were the most significant in the medium-dose BLM group. The skin collagen volume fraction (CVF) exhibited a dose-dependent increase in response to BLM administration (**Figure 2**; **Table 1**).

**Table 1:** Skin and lung pathology scores of tree shrews in each group.

Groups	n	Skin thickness (μm)	Skin inflammation score	Skin CVF (%)	Skin HYP content (mg/g)	Lung inflammation score	Lung fibrosis Ashcroft	Lung HYP content (mg/g)
Control	6	134.87±19.97	0.40±0.13	32.45±8.37	0.60±0.18	0.38±0.14	3.87±0.50	0.84±0.22
Low-dose BLM	7	152.94±14.37	0.49±0.16	36.92±4.05	0.97±0.31^*^	0.69±0.28	4.27±0.40	0.85±0.07
Medium-dose BLM	7	230.05±44.42^**#^	2.23±0.84^**##^	43.69±8.70^*^	1.21±0.22^**^	1.51±0.34^***###^	4.88±0.85^**^	0.82±0.13
High-dose BLM	9	236.21±57.02^**##^	1.49±0.35^***###^	50.29±7.45^***##^	1.29±0.35^***#^	0.91±0.32^**&&&^	4.55±0.52^*^	0.80±0.11
F		11.750	24.06	8.449	8.122	18.449	3.394	0.185
*P*		<0.001	<0.001	<0.001	<0.001	<0.001	0.033	0.906

Inflammation and fibrosis scores were significantly higher in the skin and lungs of the tree shrew SSc model. Data are shown as the mean ± SD. (1) ^*^P<0.05, ^**^P<0.01, ^***^P<0.001 compared with control group; (2) ^#^P<0.05, ^##^P<0.01, ^###^P<0.001 compared with Low-dose BLM group; (3) ^&^P<0.05, ^&&^P<0.01, ^&&&^P<0.001 compared with Medium-dose BLM group.

#### 3.2.2 Lung

The pathology of the lung in the control group was normal. Tree shrews treated with BLM showed significant pulmonary septal thickening, fibrosis and inflammatory cells infiltration (**Figure 2**). In addition, some small blood vessels showed wall thickening, lumen narrowing and occlusion. Compared with the controls, the lung inflammation scores of tree shrews in each BLM group were increased significantly in a dose-dependent manner. The Ashcroft lung fibrosis score was highest in tree shrews from medium-dose BLM group. However, no significant difference was observed in the lung hydroxyproline content among the groups (**Figure 2**; **Table 1**).

#### 3.2.3 Esophagus

HE staining showed that the esophagus of the tree shrews in each groups was structurally intact, and no inflammation or vascular changes were seen. However, Masson staining showed that fibrosis was found in all layers of esophageal tissue of some tree shrews in both the medium- and high-dose BLM groups, but not in the low-dose BLM group (**Figure 2**).

#### 3.2.4 Kidney

No inflammation, fibrosis, or characteristic vascular changes were observed in the kidney tissues in all groups of tree shrews (**Figure 2**).

### 3.3 Expression of α-SMA in skin tissues of BLM-induced SSc tree shrew model

**Figure 3:**
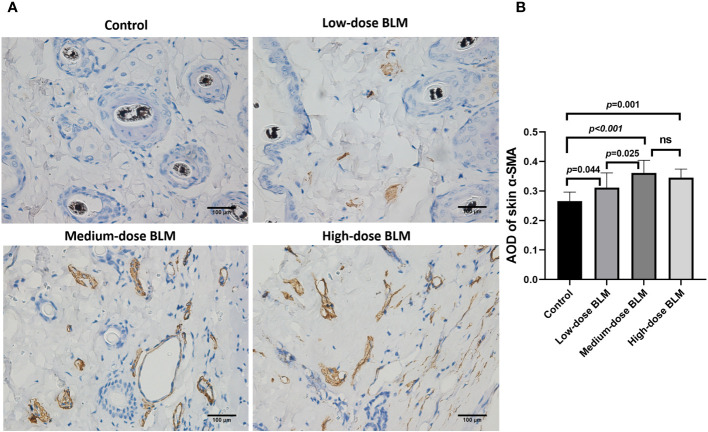
Immunohistochemical staining of α-SMA in the skin of tree shrews. The expression of skin α-SMA was significantly increased in tree shrew SSc model. **(A)** Representative immunohistochemical staining of skin α-SMA in tree shrews from each group (original magnification ×400). **(B)** The bar graph shows the average optical density value (AOD) of skin α-SMA in each group (n= 7-9 for each BLM-induced SSc tree shrew group. n =6 for the control group). ns, no significant difference.

α-SMA is one of the markers to evaluate the degree of fibrosis. The result of immunohistochemistry showed that increased expression of α-SMA in the skin tissues of tree shrews treated with BLM. The AOD value of skin α-SMA was significantly higher in each BLM-induced groups compared to the control group, expecially in medium-dose BLM group (**Figure 3**).

### 3.4 Expression of autoantibodies in BLM-induced tree shrew SSc model

**Figure 4:**
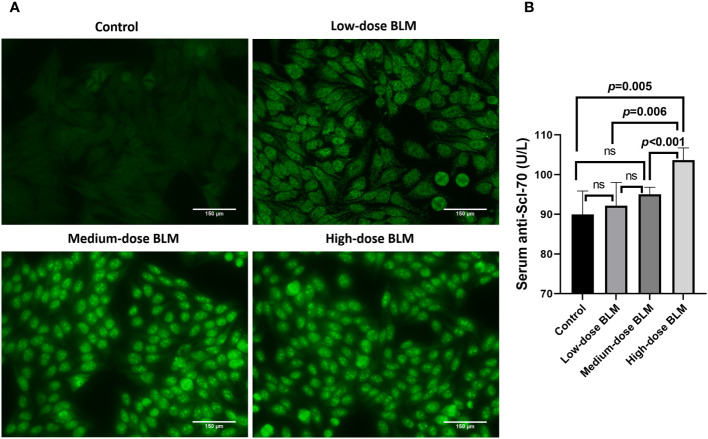
Expression of serum autoantibodies in BLM-induced SSc tree shrews model. Autoantibodies could be detected in BLM-induced tree shrew SSc model. **(A)** Expression of serum ANA in tree shrews in each group (original magnification ×200). **(B)** Bar graph showing the expression of serum anti-Scl-70 antibody in tree shrews in each group (n= 7-9 for each BLM-induced tree shrew group. n=6 for the control group). ns, no significant difference.

ANA and anti-Scl-70 antibody were detectable in serum of BLM-induced tree shrew SSc model. ANA was detected positive in only 1 tree shrew in each BLM induced group, and the rest of the animals tested negative. In the low-dose BLM induced group, ANA was weakly positive in a speckled pattern (1:100), whereas in the medium- and high-dose groups, ANA was positive in a nucleolar pattern (1:320) (**Figure 4A**). On the other hand, the anti-Scl-70 antibody titers showed a trend of BLM dose-dependent increase, which was significantly higher in the high-dose BLM group, but not statistically different in the low- and medium-dose BLM groups compared with the control group (**Figure 4B**).

### 3.5 Identification of DEGs in skin of BLM-induced SSc tree shrew model

**Figure 5:**
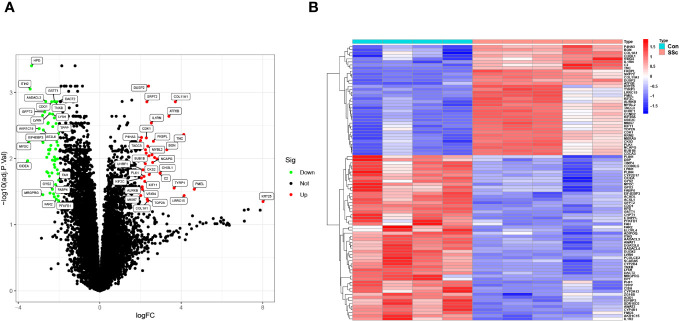
DEGs between BLM-induced SSc tree shrew and control skin samples are shown in **(A)** a volcano plot and **(B)** a heatmap.

To screen functional genes involved in the progression of SSc, we utilized the “limma” package using 5 skin samples with fibrotic lesions from the medium-dose BLM induced SSc tree shrew models and 4 from controls. A total of 90 DEGs were identifined: 54 genes were significantly downregulated, suggesting their potential role in the pathogenesis of SSc through negative regulation. Conversely, 36 genes were significantly upregulated, indicating their potential importance in the disease process through positive regulation (**Figures 5A, B**).

### 3.6 GO and KEGG enrichment analysis

**Figure 6:**
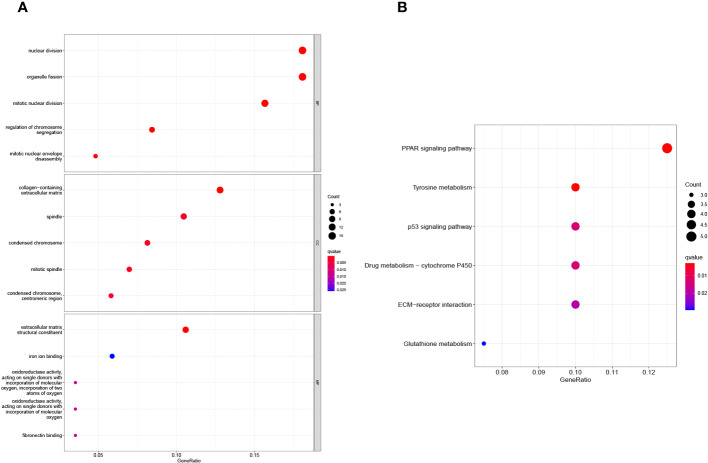
**(A)** GO and **(B)** KEGG enrichment analysis of 90 DEGs.

Using clusterProfiler software, we performed GO and KEGG pathway enrichment studies to investigate the possible biological role of shared DEGs. As shown in **Figure 6A**, GO assays revealed that 90 DEGs were mainly associated with nuclear division, organelle fission, mitotic nuclear division, collagen-containing extracellular matrix, spindle, condensed chromosome, extracellular matrix structural constituent and iron ion binding. In addition, KEGG assays suggested that 90 DEGs were mainly enriched in the PPAR signaling pathway, tyrosine metabolism, p53 signaling pathway, drug metabolism-cytochrome P450, ECM-receptor interaction and glutathione metabolism (**Figure 6B**). The research findings indicate that DEGs in SSc are mainly associated with nuclear division, extracellular matrix regulation, signaling pathways and metabolic pathways.

### 3.7 Immune infiltration in skin of BLM-induced SSc tree shrew model

**Figure 7:**
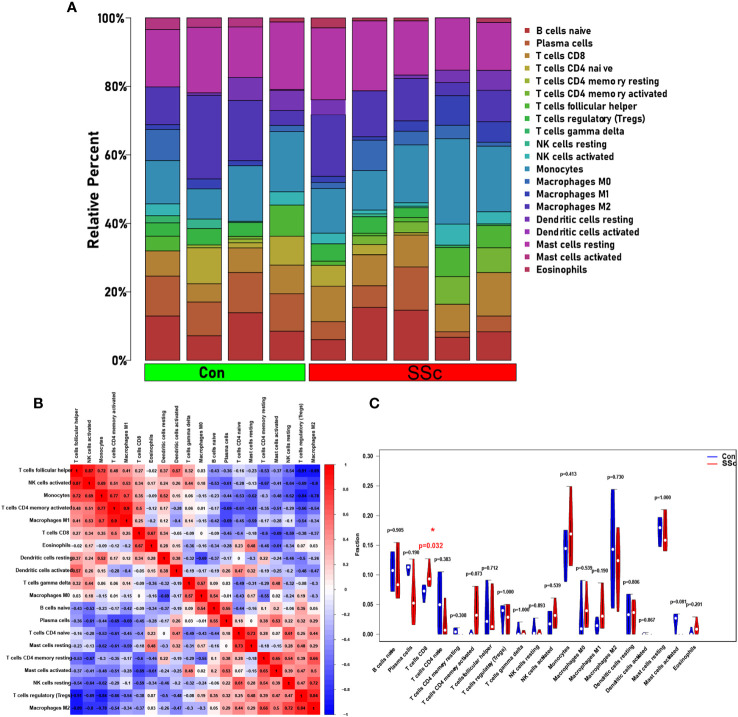
Immune infiltration in skin of BLM-induced SSc tree shrew model. **(A)** A heatmap was generated to visualize the expression levels of 20 distinct immune cell types across four control samples and five systemic sclerosis samples. **(B)** To assess the matrix encompassing 20 distinct immune cell categories, Pearson correlation coefficient analysis was performed. **(C)** The immune cell compositions between BLM-induced SSc tree shrew and control skin samples.

Based on CIBERSORT deconvolution, we identified 20 distinct types of immune cells that infiltrated in skin of BLM-induced SSc tree shrew models and controls (**Figure 7A**). The composition and proportion of immune cells in tree shrew skin were similar to that observed in human skin (data not shown). Furthermore, we established potential correlations between the different types of immune cells infiltrating the skin lesions (**Figure 7B**). M1 macrophages exhibited positive correlations with monocytes (r = 0.7) and activated CD4 memory T cells (r = 0.9), while displaying a negative correlation with plasma cells (r = -0.69). M2 macrophages demonstrated positive correlations with Tregs (r = 0.84) and resting NK cells (r = 0.72), but exhibited negative correlations with T follicular helper cells (r = -0.89), activated NK cells (r = -0.8), and monocytes (r = -0.78). Monocytes displayed positive correlations with T follicular helper cells (r = 0.72), activated NK cells (r = 0.69), and activated memory CD4 T cells (r = 0.77). Tregs demonstrated negative correlations with T follicular helper cells (r = -0.91), activated NK cells (r = -0.69), and monocytes (r = -0.84). Notably, our findings revealed a significant increase in CD8 T cells infiltration in skin of SSc tree shrew model, while activated CD4 memory T cells showed a tendency to increase in SSc tree shrew model, but the difference was not statistically significant (**Figure 7C**).

### 3.8 PPI network for the identification of the top 10 hub genes

**Figure 8:**
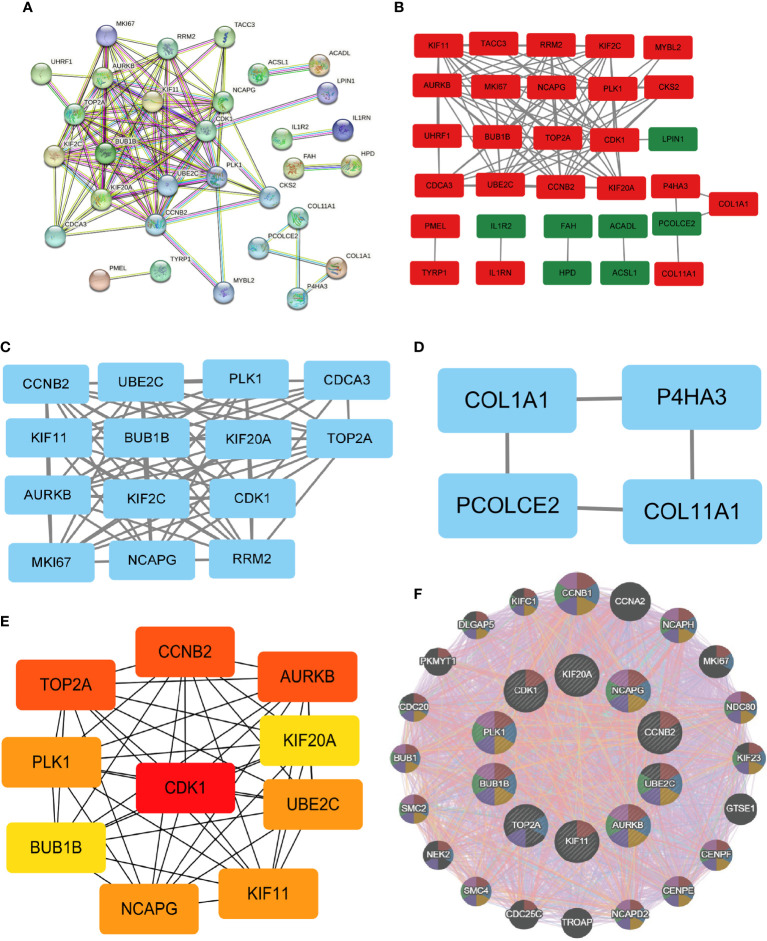
PPI network and identification of 10 hub genes. **(A, B)** Dissecting the PPI web. When the PPI was reduced to only the linked nodes, 32 genes remained. Network visualization was performed in Cytoscape 3.6.1. **(C, D)** Subgroup analysis of 32 genes. **(E)** Ten hub genes were identified. **(F)** GeneMANIA was used to examine hub genes and the genes with which they were coexpressed.

The PPI network data obtained from the STRING database was subjected to analysis using the Cytoscape software to gain insights into the key genes associated with systemic sclerosis (SSc). The analysis revealed a PPI network comprising 110 nodes, with an average local clustering coefficient of 0.461 and a combined score exceeding 0.9 (**Figure 8A**). Subsequently, the cytoHubba’s Maximum Clique Centrality (MCC) method was employed to identify the top 31 genes with the highest enrichment rating (**Figure 8B**). The subgroup analysis results are presented in **Figures 8C, D**. Notably, a total of 10 hub genes, namely KIF20A, KIF11, UBE2C, BUB1B, CDK1, CCNB2, AURKB, TOP2A, PLK1, and NCAPG, were identified (**Figure 8E**). Furthermore, GeneMANIA analysis provided insights into the relationship between these 10 core genes and their closest relatives (**Figure 8F**).

### 3.9 Functional enrichment analysis of the 10 hub genes

**Figure 9:**
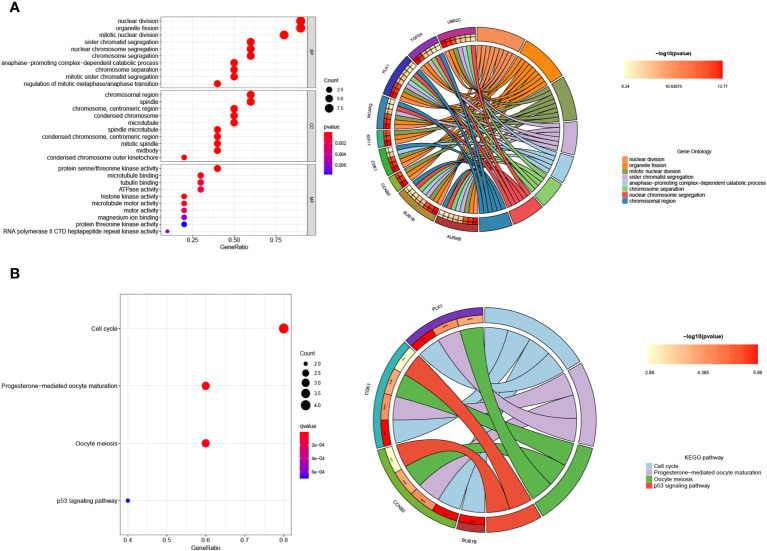
Enrichment analysis of the hub genes. **(A)** GO and **(B)** KEGG enrichment analysis of the hub genes. The significant P value of the pathway to which a gene is related is shown to the left of its outermost circle, while the gene name appears in the rightmost circle.

Functional enrichment analysis was conducted to gain insights into the potential roles of the 10 hub genes. Gene Ontology (GO) analysis revealed significant associations between these genes and various biological processes, including nuclear division, organelle fission, mitotic nuclear division, chromosomal region, chromosome, spindle, centromeric region, protein serine/threonine kinase activity, tubulin binding, and microtubule binding (**Figure 9A**). Furthermore, Kyoto Encyclopedia of Genes and Genomes (KEGG) assays demonstrated that PLK1, CDK1, CCNB2, and BUB1B were predominantly enriched in the p53 signaling pathway, oocyte meiosis, cell cycle, and progesterone-mediated oocyte maturation (**Figure 9B**).

### 3.10 The expression pattern of the 10 hub genes and their correlation with immune cells

**Figure 10:**
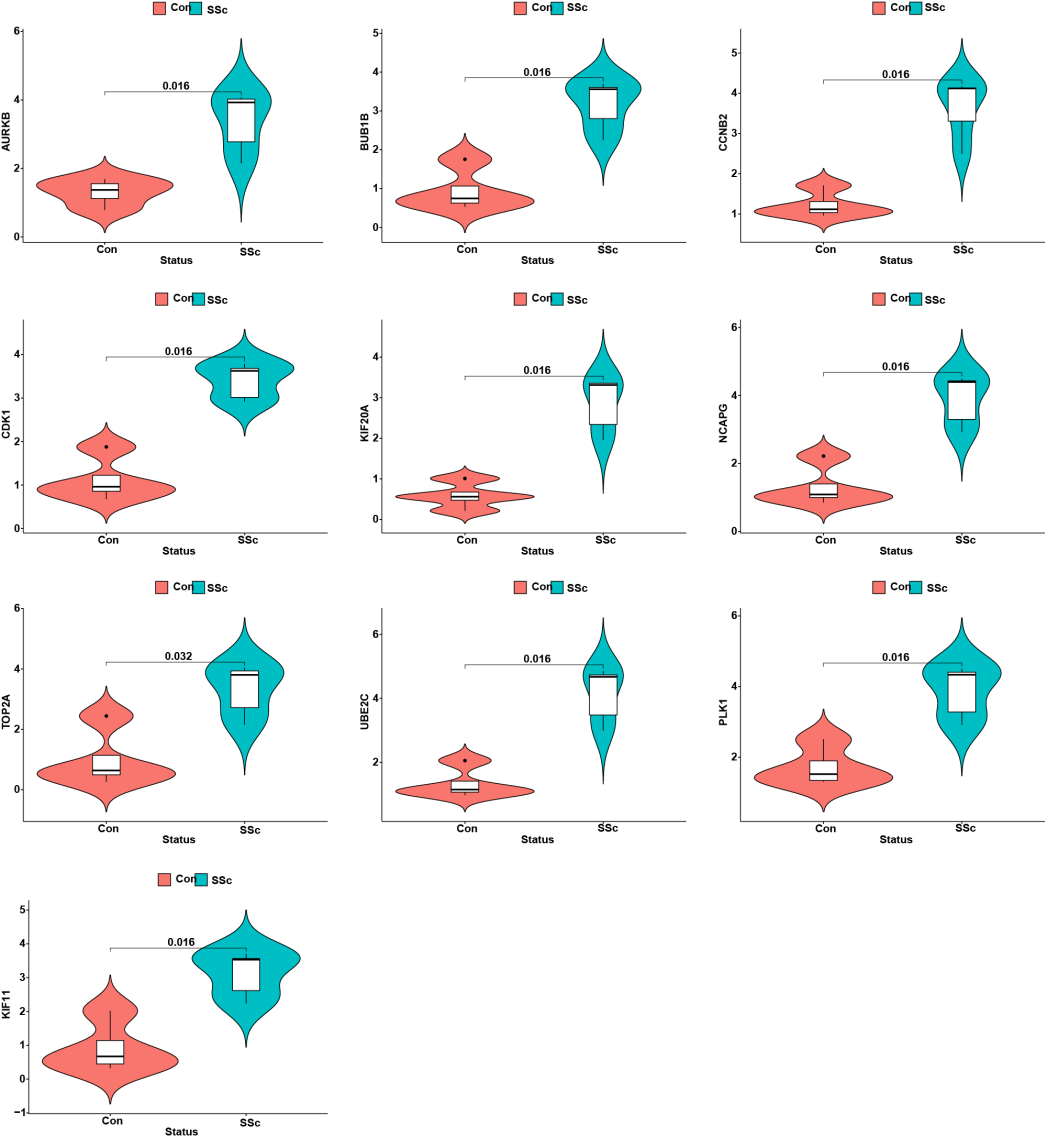
The expression patterns of 10 hub genes in skin samples of BLM-induced SSc tree shrews and controls.

**Figure 11:**
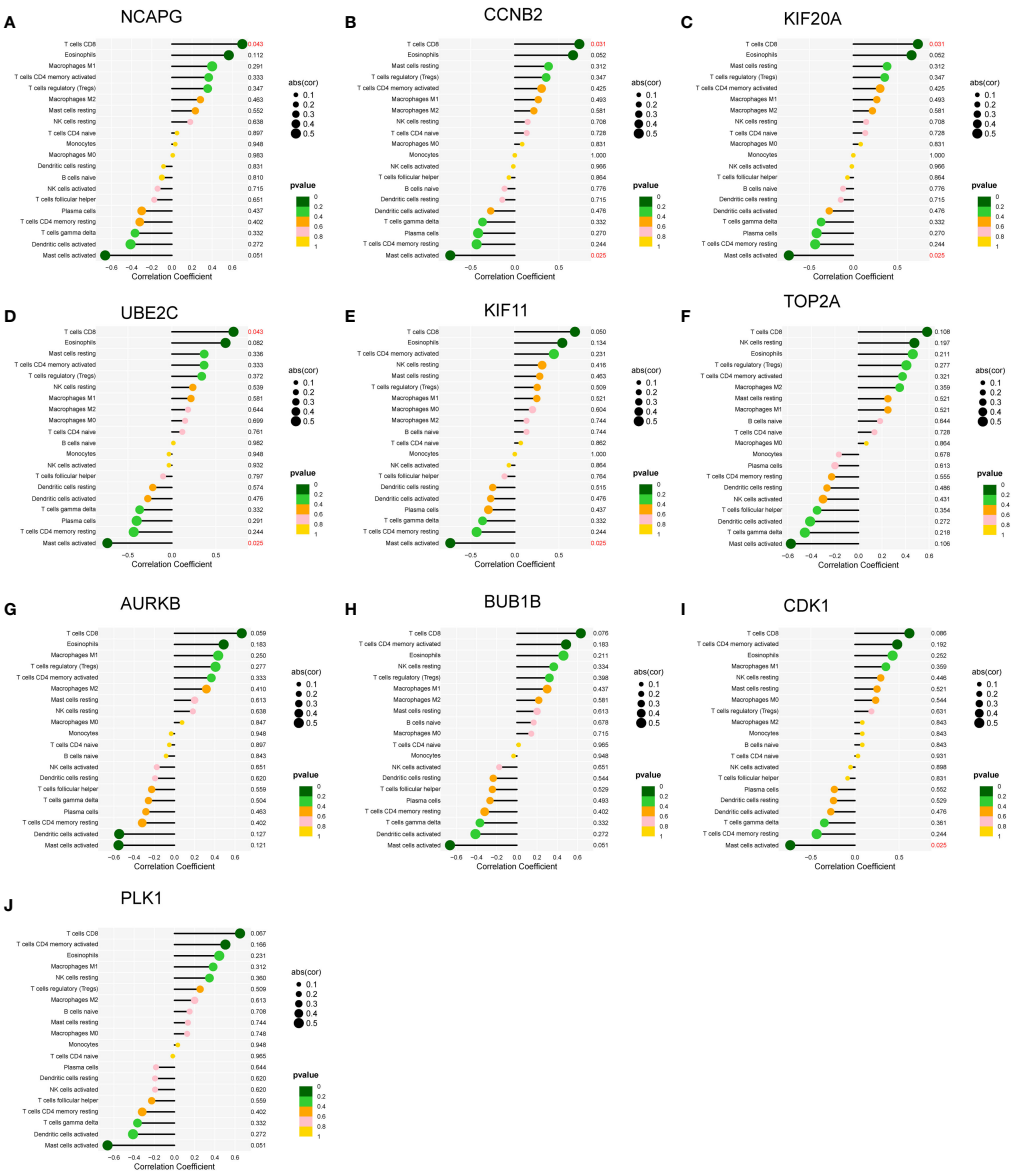
Correlations between **(A–J)** the 10 hub genes and infiltrating immune cells in skin samples of BLM-induced SSc tree shrews.

We assessed the expression patterns of the 10 hub genes in both SSc and control tree shrew skin samples. Notably, we observed a distinct upregulation of these hub genes in the skin samples of BLM-induced SSc model tree shrews compared to control samples (**Figure 10**). Furthermore, we conducted an analysis to determine the correlation between the 10 hub genes and skin-infiltrating immune cells (**Figure 11**). A significant correlation was defined as | r | > 0.4 and p < 0.05. Our results revealed a positive association between the levels of NCAPG, CCNB2, KIF20A, and UBE2C with the levels of CD8 T cells (**Figures 11A–D**). Moreover, the levels of CCNB2, KIF20A, UBE2C, KIF11, and CDK1 exhibited a negative correlation with the levels of activated mast cells (**Figures 11B, C, E, I**). However, no significant associations were observed between the other genes and immune cells (**Figures 11G–J**).

### 3.11 Conservation analysis of the 10 hub genes

To investigate the conservation of genes in tree shrew, we performed multiple sequence alignments of these 10 gene sequences in tree shrews, humans, and mice. The results showed that these 10 genes had high conservation among above species, that is, their sequences were relatively consistent among tree shrews, humans, and mice. This suggests that these genes may play important functional roles during evolution and that their sequences are conserved across species (**Supplementary Data: Excel Table 1**).

## 4 Discussion

In this study, we successfully constructed a BLM-induced SSc tree shrew model for the first time. The recommended method of modeling is subcutaneous injection of 100µl of 2 mg/L BLM solution daily for 21 days. The SSc tree shrew models exhibited inflammation and fibrosis in skin and lung, part of the animals developed esophageal fibrosis and autoantibodies, sharing similar characteristics as human SSc. Hydroxyproline and α-SMA are recognized markers of tissue fibrosis. The skin hydroxyproline content and α-SMA expression were significantly elevated in the SSc tree shrew models, providing further confirmation of the successful establishment of the model.

Autoantibodies serve as serological indicators of SSc (37). ANA is detectable in the majority of SSc patients, with the nucleolar pattern being the most prevalent. Anti-Scl-70 is a marker for diffuse SSc and is associated with rapid progression of skin lesions and pulmonary fibrosis (38). In our study, positive ANA with nucleolar pattern was detected in the BLM-induced SSc tree shrew model, although its positivity rate was low, it presents the serologic characteristics of human SSc. Similarly, higher anti-Scl-70 titers were also observed in our SSc tree shrew model. Of note, the anti-Scl-70 antibody titers in control tree shrews was higher than expected. This high background of anti-Scl-70 antibody in controls may due to the fact that the anti-Scl-70 antibody ELISA kit was not designed specifically for tree shrew but for mouse. Development of tree shrew-specific reagents will be beneficial for further research on tree shrews.

RNA sequencing was applied to comprehensively understand the genetic changes in the skin of tree shrews with SSc. Skin tissues were collected from 5 SSc tree shrew models and 4 control individuals, followed by RNA sequencing. A total of 90 DEGs were identified between the two groups. GO and KEGG enrichment analyses unveiled that these 90 DEGs exhibited significant associations with nuclear division, extracellular matrix, and were enriched in the PPAR signaling pathway, tyrosine metabolism, p53 signaling pathway, and ECM-receptor interaction. The excessive production of extracellular matrix serves as the fundamental basis of SSc, and the regulation of extracellular matrix formation remains a focal point in SSc research. Previous studies have demonstrated the crucial role of the peroxisome proliferator-activated receptor (PPAR) signaling pathway in inflammation, fibrosis, and vascular remodeling (39). Impaired PPAR-γ activity in SSc may contribute to the uncontrolled progression of fibrosis and pulmonary arterial hypertension (40, 41). Notably, the pan-PPAR agonist lanifibranor has been shown to mitigate lung fibrosis and cardiorespiratory manifestations in a mouse model of SSc (42). Transcriptome analysis of lung tissues afflicted with SSc-associated interstitial lung disease has revealed significant enrichment of the p53 signaling pathway at both the tissue and cellular levels, which is closely associated with lung function, cellular senescence, and apoptosis (43). Further investigations into the functions and regulatory mechanisms of these genes hold promise for a deeper understanding of the pathogenesis of SSc.

The analysis of immune cells in tree shrews is impeded by the limited availability of commercial experimental reagents. Our attempts to detect immune cells in tree shrews using flow cytometry with either human or murine flow cytometry antibodies yielded unsuccessful results (data not shown). Consequently, we employed CIBERSORT to analyze the immune cell composition. In the skin tissues of both groups of tree shrews, we identified 20 types of immune cells, and their composition and proportions resembled those observed in human skin (data not shown). Notably, we observed dysregulated levels of CD8 T cells in SSc model specimens. Consistent with our findings, CD8 T cells were found to be enriched in the skin of SSc patients and have been implicated in the pathogenesis of SSc through their proinflammatory, profibrotic, and cytotoxic effects (44–46).

To further elucidate the key genes involved in the progression of SSc, we conducted PPI network analysis and identified 10 hub genes: KIF20A, KIF11, UBE2C, BUB1B, CDK1, CCNB2, AURKB, TOP2A, PLK1, and NCAPG. We confirmed that the expression of these 10 hub genes was significantly upregulated in SSc samples compared to normal skin samples. To investigate the conservation of these 10 key genes in different species, we performed multiple sequence alignments of these 10 gene sequences in tree shrews, humans, and mice. The results showed that these 10 genes had high conservation among different species, that is, their sequences were relatively consistent among tree shrews, humans, and mice. This suggests that these genes may play important functional roles during evolution and that their sequences are conserved across species.

Among these proteins, PLK1, CDK1, TOP2A, and AURKB are protein kinases that play crucial roles in regulating cellular physiological processes, including the cell cycle, cell division, and signaling. Importantly, these kinases are implicated in the pathogenesis of fibrosis. A comprehensive analysis of lung samples from 585 patients with idiopathic pulmonary fibrosis (IPF) revealed that CDK1, a cell cycle-dependent kinase, is a key regulator in fibrosis (47). CDK1 regulates fibrosis by controlling the stemness and self-renewal of pathological mesenchymal progenitor cells in IPF (48). Inhibition of CDK activity in SSc hinders fibroblast proliferation and directly suppresses matrix production (49). VCE-004.8, a synthetic cannabidiol derivative that lacks psychotropic effects, has been shown to prevent and inhibit angiotensin II-induced fibrosis in mice by suppressing the expression of CDK1 and TOP2A (50). However, the function of TOP2A, a cell cycle regulatory protein, in SSc or fibrosis remains unexplored. PLK1, also known as Polo-like kinase 1, interacts with CDK1 to ensure proper cell division and orderly replication. PLK1 has been predicted to be a marker gene in IPF and is predominantly expressed in myofibroblasts. PLK1 serves as a promoter of myofibroblast differentiation. *In vitro* experiments have demonstrated that suppression of PLK1 leads to a reduction in α-SMA expression and inhibition of TGF-β1-mediated myofibroblast differentiation (51). *In vivo* studies have shown that inhibition of PLK1 alleviates pulmonary fibrosis induced by BLM in mice by inhibiting the proliferation of pulmonary fibroblasts (52, 53).

AURKB, also known as Aurora kinase B, is upregulated in fibroblasts from IPF patients and in lung tissues of TGF-α and BLM-induced pulmonary fibrosis. AURKB promotes the proliferation and survival of fibroblasts, and thus, inhibition of AURKB attenuates fibroblast activation and lung fibrosis (54). Further investigation into the functions and regulatory mechanisms of these five genes may provide insights into the pathological processes underlying SSc. UBE2C is a gene associated with cellular senescence and has the potential to serve as a biomarker for poor prognosis in IPF patients (55). However, functional studies regarding UBE2C have not yet been conducted. To date, there have been limited reports on the relationships between CCNB2, NCAPG, KIF20A, KIF11, and BUB1B with SSc or fibrosis. However, our findings indicate that the levels of NCAPG, CCNB2, KIF20A, and UBE2C are positively correlated with CD8 T cell levels, suggesting their potential role in promoting the progression of SSc by positively regulating CD8 T cells. Nevertheless, further research is warranted to fully elucidate these mechanisms.

## 5 Conclusion

This study successfully established an SSc model in tree shrews, demonstrating similar pathological and serological changes to human SSc. Through RNA-seq analysis, we identified 90 differentially expressed genes (DEGs) in the skin of SSc models compared to controls, providing insights into the transcriptome profiles of tree shrews. Additionally, we investigated the composition of infiltrating immune cells in the skin of SSc models and identified 10 key genes, including KIF20A, KIF11, UBE2C, BUB1B, CDK1, CCNB2, AURKB, TOP2A, PLK1, and NCAPG, and were further confirmed to be highly conserved in tree shrews, humans and mice. These findings contribute to a better understanding of the underlying mechanisms involved in the development of systemic sclerosis. The dysregulated expression of these key genes may contribute to disease progression. Overall, our research provides valuable guidance for future investigations and clinical interventions in systemic sclerosis. However, despite these significant discoveries, further studies are needed to fully comprehend the complexity of this disease.

## Data availability statement

The raw sequencing data of this present study have been deposited in the Genome Sequence Archive in BIG Data center (https://bigd.big.ac.cn/), Beijing Institute of Genomics, Chinese Academy of Science, under the accession number: CRA012348.

## Ethics statement

The animal study was approved by the Experimental Animal Ethics Committee of Guangxi Medical University (No. 20180521). The study was conducted in accordance with the local legislation and institutional requirements.

## Author contributions

LZ: Data curation, Funding acquisition, Methodology, Project administration, Writing – original draft. SC: Data curation, Methodology, Writing – original draft. QW: Data curation, Methodology, Software, Writing – original draft. XL: Methodology, Writing – original draft. WZ: Methodology, Writing – original draft. FD: Data curation, Writing – original draft. WA: Methodology, Writing – original draft. FQ: Methodology, Writing – original draft. LL: Writing – review & editing. CZ: Conceptualization, Funding acquisition, Supervision, Writing – review & editing.
